# Chemical composition of *Origanum majorana, Mentha* sp*icata* and *Ocimum basilicum* essential oils and their impact on *Spodopteralittoralis*: toxicity and immune response

**DOI:** 10.3389/fpls.2025.1737742

**Published:** 2026-01-12

**Authors:** Mofeed Askar, Elsayed E. Hafez, Ahmed A. Saleh, Amal H. Marei, Hanaa S. Hussein, El-Seedi H. R., Fatma H. Galal, Yaohai Li, Honghua Su

**Affiliations:** 1 Plant Protection College, Yangzhou University, Yangzhou, Jiangsu, China; 2 Plant Protection Department, Faculty of Agriculture, Damietta University, Damietta, Egypt; 3 City of Scientific Research and Technology Applications, Arid Lands Cultivations Research Institute, New Borg El-Arab, Alexandria, Egypt; 4 Animal and Fish Production Department, Faculty of Agriculture (Al-Shatby), Alexandria University, Alexandria, Egypt; 5 Department of Applied Entomology and Zoology, Faculty of Agriculture, Alexandria University, Alexandria, Egypt; 6 Department of Chemistry, Faculty of Science, Menoufia University, Shebin El-Kom, Egypt; 7 Department of Biology, College of Science, Jouf University, Sakaka, Saudi Arabia; 8 Xizang Institute of Forest Trees, Lhasa, Xizang, China

**Keywords:** defense gene, essential oils, M. spicata, O. basilicum, O. majorana, Spodoptera littoralis, toxic chemical

## Abstract

**Introduction:**

This study presents the essential oils (EOs) derived from *Origanum majorana* L. (Marjoram), *Mentha spicata* L. (Spearmint) and *Ocimum basilicum* L. (Basil), which are explored for their insecticidal potential against *Spodoptera littoralis* larvae.

**Methods:**

EOs were extracted and analyzed using gas chromatography-mass spectrometry (GC-MS), identifying 47, 37 and 27 compounds in *O. majorana, M. spicata* and *O. basilicum*, respectively.

**Results:**

Major constituents include terpinen-4-ol (25.47 %) and sabinene (18.3%) in *O. majorana*, piperitenone oxide (43.83 %) in *M. spicata*, and methyl (E)-cinnamate (48.69 %) in *O. basilicum*. Toxicity assays demonstrated significant larvicidal activity with LC_50_ values of 1.18 % for *O. majorana*, 0.43% for *M. spicata*, and 0.51% for *O. basilicum*. Furthermore, these EOs notably influenced the expression of defense-related genes in *S. littoralis*, with treated larvae showing an ~80% increase in *PR1*, endoglucanase (*PR2*) and chitinase gene expression compared to controls. Differential display confirmed the amplification of these regulated genes in treated insects.

**Discussion:**

This research underscores the efficacy of EOs from *O. majorana, M. spicata* and *O. basilicum* as natural insecticides, providing valuable insights for sustainable pest management through specific gene markers.

## 1 Introduction

The cotton leafworm, *Spodoptera littoralis* (Lepidoptera: Noctuidae), is a significant polyphagous pest causing substantial economic losses across over 40 plant families, including key crops such as cotton, tomatoes, and beans (Adam et al., 2019; ElShahed et al., 2023). In Egypt, it is the primary pest of cotton (El-Sayed et al., 2020). The economic impact of this pest is well-documented, necessitating effective control measures. Traditionally, synthetic insecticides like organophosphates and pyrethroids have been used to control *S. littoralis*, but their extensive use has led to the development of pest resistance and raised serious environmental concerns (Ahmad et al, 2009; Ismail, 2023; Ismail, 2024). Consequently, research is shifting toward ecologically friendly alternatives within Integrated Pest Management (IPM) frameworks, with increasing interest in botanicals and microorganisms (Aydin and Gürkan, 2006; Siddiqui et al., 2023; Pu and Chung, 2024).

Plant-derived products are critical to IPM strategies aimed at reducing reliance on synthetic pesticides. Essential oils (EOs) derived from aromatic plants show promise as low-risk insecticides, effectively managing pest species through neurotoxic effects and the inhibition of insect enzymes such as acetylcholinesterase (AChE) (Dougoud et al., 2019; Singh et al, 2019; Tawfeek et al., 2021; Mahawer et al., 2024). Recent field studies on *S. littoralis* confirm that the modulation of enzymes like AChE and Glutathione S-Transferase (GST) is a critical mode of action for bio-rational insecticides, affecting the insect’s nervous system and detoxification capabilities. Beyond neurotoxicity, a key mechanism of EO action is the induction of oxidative stress. Insects possess antioxidant defense enzymes like superoxide dismutase (SOD) and catalase (CAT) to manage oxidative radicals. Research shows that EOs can disrupt this balance, inhibiting these enzymes and causing cellular damage, which contributes to their insecticidal efficacy (Krishnan and Kodrík, 2006).

The studies in the field of pest management often emphasizes the identification of effective plants within the Lamiaceae family, recognized for their high EO production and established insecticidal properties (Inanoglu et al., 2023; Kowalczyk et al., 2023). Commonly studied genera include *Mentha*, *Ocimum* and *Origanum*, with a focus on widely available species that demonstrate proven efficacy and economic viability for agricultural practices (Inanoglu et al., 2023; Kowalczyk et al., 2023). For instance, *Origanum majorana* EO, rich in monoterpenoids, has demonstrated toxicity against *S. littoralis* larvae and the ability to inhibit detoxifying and antioxidant enzymes like GST and SOD (Krishnan and Kodrík, 2006; Inanoglu et al., 2023; Kowalczyk et al., 2023).

Among these species, *Origanum majorana* is recognized for its insecticidal properties (Kakouri et al., 2022), while *Mentha* sp*icata* is noted for its antimicrobial and insecticidal capabilities (Bendifallah et al., 2020). Additionally, *Ocimum basilicum* demonstrates considerable antibacterial and insecticidal effects against various pests (Politeo et al, 2007; Worku et al., 2024). Despite the progress in studying EO insecticidal properties, significant gaps remain. The existing literature has thoroughly explored the chemical composition, contact toxicity, and some biochemical impacts (e.g., on detoxification and antioxidant enzymes) of various EOs against *S. littoralis*. However, the effects on the insect’s *immune response* at the molecular level are poorly understood. Immune and developmental processes in insects are regulated by specific genes and enzymes. For example, the enzyme chitinase is crucial for molting and development, and its disruption can impair insect growth. Furthermore, immune pathways involving genes like β-1, 3-glucan-binding protein (βGBP) are vital for defense against pathogens and parasitoids. Investigating whether EOs suppress such immune-related genes (*PR1*, *PR2*, *chitinase*) would reveal a novel, sublethal mode of action that weakens the insect’s overall fitness and defense capacity, a dimension largely unaddressed in prior research (Krishnan and Kodrík, 2006; Politeo et al, 2007; Govindarajan et al., 2013; Rodríguez-González et al., 2019; Kowalczyk et al., 2023; Ismail, 2024; Worku et al., 2024).

This study aimed to characterize the chemical composition of EOs from *O. majorana*, *M.* sp*icata*, and *O. basilicum* and to evaluate their larvicidal effects on third instar larvae of *S. littoralis*. Based on their superior toxicity, the EOs of *O. basilicum* and *M.* sp*icata* were selected to further investigate the immune response mechanisms of *S. littoralis*, contributing insights into their dual roles as larvicidal agents and modulators of insect immunity.

## 2 Materials and methods

### 2.1 Chemicals used in the study

In this study, a variety of chemicals and reagents were employed to ensure the accuracy and reliability of experimental procedures. Hexane (Product Code: 12352001, MilliporeSigma, USA; https://www.carolina.com/catalog/detail.jsp?prodId=873400) served as the primary solvent for extracting EOs and creating oil dilutions. Lambda-cyhalothrin (Analytical Standard, PESTANAL^®^, Sigma-Aldrich) was used as a positive control insecticide. Anhydrous Magnesium Sulfate (MgSO_4_, Product Code: 87-3400, American Chemical Society, USA) was used to dry the hexane extracts, ensuring the oils were free of moisture. β-Mercaptoethanol (Product Code: M6250/MFCD00004890, Sigma Aldrich-MERK, Germany; https://www.sigmaaldrich.com) was incorporated into the RLT buffer to prevent oxidative degradation of RNA during extraction. To sustain laboratory populations, a 20% sucrose solution was prepared in-house using sucrose from Sigma-Aldrich (Product Code: S7903, USA) to provide essential nourishment for adult *S. littoralis*. In addition to these specific chemicals, other general chemicals for this study were sourced from Becton Dickinson (Sparks, MD, USA) and Loba Chemie PVT. LTD (Mumbai, India), with further chemicals obtained from Sigma-Aldrich (St. Louis, MO, USA).

### 2.2 Insects rearing

A laboratory-susceptible strain of the cotton leafworm *S. littoralis* (Boisduval) (Lepidoptera: Noctuidae) was acquired from the Agricultural Research Centre in Dokki, Giza, Egypt. Larvae were maintained under constant conditions in the laboratory at 28 ± 2 °C and 65% relative humidity and were reared on common bean leaves (*Phaseolus vulgaris*) (Malpighiales: Euphorbiaceae) until pupation. Pupae were individually housed in jars with muslin covers secured by rubber bands at the neck. The emerged adults were provided with a 10% sucrose solution for sustenance.

### 2.3 Essential oil extraction

Leaves of *O. majorana*, *M.* sp*icata* and *O. basilicum* were harvested during the flowering stage in Spring 2023 from the Research Centre for Medicinal and Aromatic Plants in El-Kanater, El-Qalyoubeyah, Egypt. The plants were cultivated under standard agricultural conditions, including regular irrigation and fertilization, and were confirmed to be free from pesticide application prior to harvest. The plant materials were authenticated by Prof. Dr. El-Seedi H.R, curator of the herbarium at the Faculty of Science, El-Menoufia University. Voucher specimens were prepared and deposited under their supervision and were assigned the unique accession numbers 2023-OM01 (O*. majorana*), 2023-MS01 (*M.* sp*icata*), and 2023-OB01 (*O. basilicum*). This documentation serves as a critical reference point for future research, providing a permanent, verifiable record of the plant materials used in the study. The specimens can be accessed for comparison or further study to confirm the identity and authenticity of the plant species involved in the EO extraction process.

The leaves were washed with distilled water and then air-dried at room temperature in the shade for 4 days. This step, commonly employed in phytochemical studies (Giacometti et al., 2018), serves to standardize the plant material by reducing moisture, prevents fungal growth during short-term storage, and facilitates grinding. The dried leaves were ground into a fine powder and immediately subjected to hydrodistillation using steam (steam distillation). This process was performed on one hundred grams of each powdered plant material for 4 hrs. The primary extraction medium was water, which carried the volatile compounds into the distillate. The resulting hydrodistillate, a mixture of water and volatile oil, was collected. To separate the essential oil from the aqueous phase and to concentrate it, the distillate was subjected to liquid-liquid extraction with 100 ml of hexane. Hexane was used specifically for this separation and concentration step due to its low polarity, high volatility, and ability to effectively partition the essential oil from water. The hexane phase, containing the essential oil, was then dehydrated with anhydrous MgSO_4_, filtered, and concentrated using a rotary evaporator at 20°C under reduced pressure (Pålsson et al., 2008). The extracted essential oils were quantified. The percentage yield was calculated on a weight per weight (w/w) basis using the formula:

]Weight of Essential Oil Obtained/Weight of Dry Plant Material[× 100.

Subsequently, stock solutions were prepared in hexane at a concentration of 20 mg/mL. All EOs were stored in tightly sealed amber glass vials at 4°C to preserve their chemical integrity until bioassay.

### 2.4 GC-MS/FID analysis of the essential oils

The analysis was conducted using a TRACE GC Ultra Gas Chromatograph (GC) (Thermo Scientific Corp., USA). The system was equipped with a dual detection setup: a mass spectrometer detector (ISQ Single Quadrupole Mass Spectrometer) for compound identification and a flame ionization detector (FID) for accurate quantification. GC was fitted with a TR-5 MS column (30 m × 0.32 mm i.d., 0.25 µm film thickness). Operational parameters included an injector temperature of 200 °C, an FID detector temperature of 200°C, and a split ratio of 1:10. For the mass spectrometer, the ionization voltage was set to 70 eV. Helium served as the carrier gas at a constant flow rate of 1.33 mL/min. To determine the Kovats Retention Indices (RIs) of the essential oil components, an n-alkane mixture (C_8_-C_20_) was co-injected under identical chromatographic conditions.

The EOs of *O. majorana*, *M.* sp*icata* and *O. basilicum* were analyzed utilizing GC-MS, with a 1 μL sample injected. The oven temperature was initially set at 60°C for 1 minute and then ramped up to 214°C at a rate of 4°C/min, maintaining this temperature for another minute. Identification of the essential oil components was conducted by comparing the mass spectra of each compound with those stored in the NIST (NIST Mass Spectrometry Data Center, 2014), Sparkman (2005) and Stein (1999) libraries. Further confirmation was achieved by comparing the calculated experimental RIs of the compounds with literature RI values. A compound was considered identified when its mass spectrum matched the library data with a similarity index >90% and its experimental RI was within an acceptable range (± 10 units) of the literature RI value.

### 2.5 Larvicidal activity bioassay

The topical larvicidal activity of the essential oils was evaluated against third-instar *S. littoralis* larvae. Final test concentrations for each EO were prepared by serial dilution in hexane, starting from their respective 20 mg/mL stock solutions (Section 2.3), to obtain the following ranges: *O. majorana* (0.5, 1.0, 2.0, 3.0, and 4.0%), *M.* sp*icata* (0.25, 0.75 and 1.5%), and *O. basilicum* (0.3, 0.7, 1.0 and 1.5%). A positive control was established using lambda-cyhalothrin, with test concentrations of 0.001, 0.005, 0.01, and 0.02% (w/v) prepared from a stock solution in hexane. For the bioassay, a volume of 1 mL of each hexane-based EO dilution or insecticide solution was spread uniformly onto the bottom of a glass Petri dish (9 cm diameter). A negative control treatment of 1 mL of pure hexane was prepared identically. The solvent was allowed to evaporate completely under a fume hood for 10 minutes, leaving a uniform film of EO residue.

Groups of ten third-instar larvae were placed in each treated Petri dish. Larvae were confined to the treated substrate for a continuous exposure period of 3 hrs. After this exposure, all surviving larvae were gently transferred to clean plastic containers (e.g., 500 mL jars) provisioned with an excess of fresh, untreated common bean leaves (*Phaseolus vulgaris*).

Larval mortality was assessed at 24, 48, 72, and 96 hrs post-initial exposure. Larvae were considered dead if they showed no movement upon gentle prodding with a soft brush. Each concentration, including the hexane control, was replicated three times (n=3 replicates of 10 larvae each). Time-mortality data were analyzed, and median lethal concentration (LC_50_) values with 95% confidence intervals were calculated using probit analysis according to Finney (1971).

### 2.6 Molecular analysis of larval defense system gene expression

The sublethal effects of essential oil treatment on the physiological defense system of *S. littoralis* were assessed by analyzing the expression of three key defense-related genes using quantitative Real-Time PCR (qRT-PCR), with the 18S rRNA gene serving as the housekeeping reference. The genes were selected for their critical roles in immune and structural defense: Pathogenesis-related proteins 1 and 2 (*PR1* and *PR2*) are central to the humoral immune response, with *PR1* exhibiting broad antimicrobial activity and *PR2* (a β-1,3-glucanase) involved in fungal cell wall degradation and immune pathway activation. Chitinase is essential for chitin metabolism, which is vital for molting, peritrophic membrane integrity, and defense against pathogens. This analysis aimed to determine whether essential oil exposure disrupts these fundamental defense mechanisms in larvae.

#### 2.6.1 Larval treatment for molecular analysis

Based on the larvicidal activity results, the essential oils of *O. basilicum* and *M.* sp*icata* were selected for gene expression analysis. Third-instar *S. littoralis* larvae were topically treated with a sub-lethal concentration (LC_25_) of each EO, prepared and applied in hexane as described in Section 2.5. A solvent control group was treated with hexane only (0% EO) to account for any potential effects of the carrier solvent. This group served as the baseline control for all gene expression analyses. Following treatment, larvae were transferred to containers with fresh common bean leaves and maintained under standard rearing conditions.

For each EO and the control, total RNA was extracted from pools of five whole larvae sampled at a post-exposure time of 24 hrs. This sampling time point was selected to capture early transcriptional changes in defence-related genes. Each treatment condition (*O. basilicum* LC_25_, *M.* sp*icata* LC_25_, and hexane control) was independently replicated three times (n = 3 biological replicates, where each replicate is one RNA pool from 5 larvae).

Subsequently, for the qRT-PCR analysis, each cDNA sample (derived from one biological replicate) was analyzed in triplicate on the qPCR plate (n = 3 technical replicates per biological replicate).

#### 2.6.2 Extraction of total RNA

The isolation of total RNA from larvae was conducted using the RNeasy kit following the manufacturer’s protocol (Qiagen, Germany). Briefly, 100 mg of tissues was homogenized in 600 µL of the lysate buffer RLT (with β-Mercapto Ethanol [β-ME] added to the buffer before use) using a mortar. After centrifugation at 14,000 rpm for 2 minutes, 400 µL of absolute ethanol was added to the cleared supernatant and thoroughly mixed by pipetting. The sample was then loaded onto a RNeasy spin column in a 2 ml collection tube and centrifuged for 1 minute at 10,000 rpm. Following discarding of the filtrate, the spin column was washed with 700 μL of buffer RW1, centrifuged for 1 minute at 10,000 rpm, and this washing step was repeated twice with 500 μL of buffer RPE. After transferring the RNeasy spin column to a new tube and a final centrifugation at full speed, the RNA was eluted by adding 40 μL of RNase-free water directly to the spin column membrane. The elution was incubated at room temperature for 1 minute, then centrifuged at 8000 rpm for 1 minute before storing the RNA at -70°C (RNeasy Mini Handbook, Qiagen, Hilden, Germany).

#### 2.6.3 Reverse transcription of the extracted RNA

The first-strand cDNA was synthesized using Moloney Murine Leukemia Virus Reverse Transcriptase Enzyme (Fermentas, USA). Reverse transcription reactions utilized the primer oligo dT primer. Each 25 µL reaction mixture contained 2.5 µL of 5X buffer with MgCl_2_, 2.5 µL of 2.5 mM dNTPs, 1 µg of primer, 2 µg of RNA, and 200 U of Reverse Transcriptase Enzyme. The RT-PCR amplification was carried out in a thermal cycler (Eppendorf, Germany) programmed at 42°C for 1 hour and 72°C for 10 minutes. Subsequently, the cDNA was stored at -20°C until further use.

#### 2.6.4 Quantitative real-time PCR assay

qRT-PCR was conducted using SYBR Green PCR Master Mix from Fermentas, USA with three specific primers (*PR1*, *PR2* and *Chitinase*) as detailed in **Table 1**. The 18S rRNA gene was used as an endogenous reference gene for normalization. This technique quantifies cDNA levels after each cycle using fluorescent dyes, with the signal increase being proportionate to the amount of PCR product generated. Each 25 μL reaction mixture consisted of 1 μL of 10 pmol/μ1 of each primer, 1 μL of template cDNA (50 ng), 12.5 μL of 2X SYBR Green PCR Master Mix, and 9.5 μL of nuclease-free water. Prior to loading the samples in the rotor wells, they were properly spun and each sample was run in triplicate.

**Table 1:** Nucleotide sequences of the primers used for qRT-PCR and differential display PCR analysis.

Technique	Target gene	Primers	Sequences (5’–3’)	Annealing °C
Real-time PCR	18sRNA	TR	AAACGGCTACCACATCCAA	
	18sRNA	RR	TGTTCAAAGTAAACGTGCCG	
	Endoglucanase 1	TR	TTCTTCCCTCGAAAGCTCAA	**60 °C**
	Endoglucanase 1	RR	GCGTACCCCAGGCTAAGTTT	**60 °C**
	Endoglucanase 2	TR	GGCCATCCACTCTCAGACACA	**60 °C**
	Endoglucanase 2	RR	TCCGGGGTATGTTATGGAAGA	**60 °C**
	Chitinasae A1	TR	GCGGATCCCAACGCACTGCAACCGATTAT	**60 °C**
	Chitinase A1	RR	GCCCATGGAAGGAATCAGTTATGCGCAAAT	**60 °C**
Differential PCR	R1	Oligo (dT)	TGCCCGTVGT	**30 °C**
	R2	Oligo (dT)	CAGGCCCTTC	**30 °C**
	R4	Oligo (dT)	ACGACCGACA	**30 °C**

The bold values indicate the unit of measurement for temperature, which is degrees Celsius (°C).

The amplification program commenced at 95 °C for 10 minutes, followed by 40 cycles including denaturation at 95°C for 15 seconds, annealing at 60°C for 30 seconds and extension at 72°C for 30 seconds. After cycling, melting curves were generated to confirm the absence of non-specific products, with data acquisition taking place during the extension step. The Rotor-Gene 6000 (QIAGEN, ABI System, USA) was used for the reaction. The PCR products were visualized on a 1.5% agarose gel and photographed utilizing a gel documentation system (Chemi.Doc™ XRS+ with Image Lab™ Software, BIO-RAD, USA).

#### 2.6.5 Quantitative real-time PCR data analysis

The relative expression ratio was accurately quantified and calculated according to Livak and Schmittgen (2001). Accordingly, for each biological sample, the difference (Δ) in quantification cycle value (C_T_) between the target (C_T (target)_) averaged from three technical repeats) and the reference (C_T (reference)_), a fixed C_T_ value was used for all samples) was first transformed into relative quantities (RQ) using the exponential function with the efficiency (E) of the PCR reaction.

The C_T_ (threshold of cycle) value of each detected gene was determined by automated threshold analysis on ABI System. The C_T_ value of each target gene was normalized to C_T (reference)_ to obtain ΔC_T (target)_ where.

ΔC_T (target)_ = (C_T (target)_ – C_T (reference)_).

ΔC_T (control)_ = (C_T (control)_ – C_T (reference)_).

The relative expression quantity of the target gene was indicated with.

ΔΔCT = (ΔC_T (target)_ – ΔC_T (control)_) according to 2^-ΔΔCt^ algorithm.

### 2.7 Statistical analysis

Mortality was assessed for each replicate as:


$$\text{Mortality rate}(\%)=\frac{\text{Number of dead insects}}{\text{Total number of treated insects}}\text{ x }100\text{ \%}$$

Where applicable, mortality data were corrected for natural mortality in the control group using Abbott’s formula (Abbott, 1925):


$$\text{Corrected mortality }(\%)=\frac{\text{Treatment mortality }-\text{ Control mortality}}{100-\text{Control mortality }}\text{ x }100\%$$

These corrected mortality values were then subjected to probit analysis (Finney, 1971) performed with POLO Plus software. For each essential oil, the hexane-only control treatment was included as the 0% concentration data point in the concentration-response model. This analysis calculated the median lethal concentrations (LC_50_) with their corresponding 95% fiducial limits (FL), slopes, and standard errors.

To determine if the toxicity (LC_50_) differed significantly among the three essential oils, a likelihood ratio test for the equality of their probit regression lines was conducted. Where the overall test indicated significant differences (*p* < 0.05), pairwise comparisons were performed. LC_50_ values were considered statistically different when their 95% fiducial limits did not overlap, and this was confirmed by the significance of the relevant pairwise tests.

For each essential oil and the synthetic insecticide, the mean percentage mortality (raw, observational data) at each time point (24, 48, 72, 96 hrs) was compared across all tested concentrations (including 0%) using one-way ANOVA at each time point, followed by Tukey’s HSD *post-hoc* test for pairwise comparisons (*p* < 0.05). Data are presented as mean ± standard error of the mean (SEM).

## 3 Results

### 3.1 Essential oil yield

**Figure 1:**
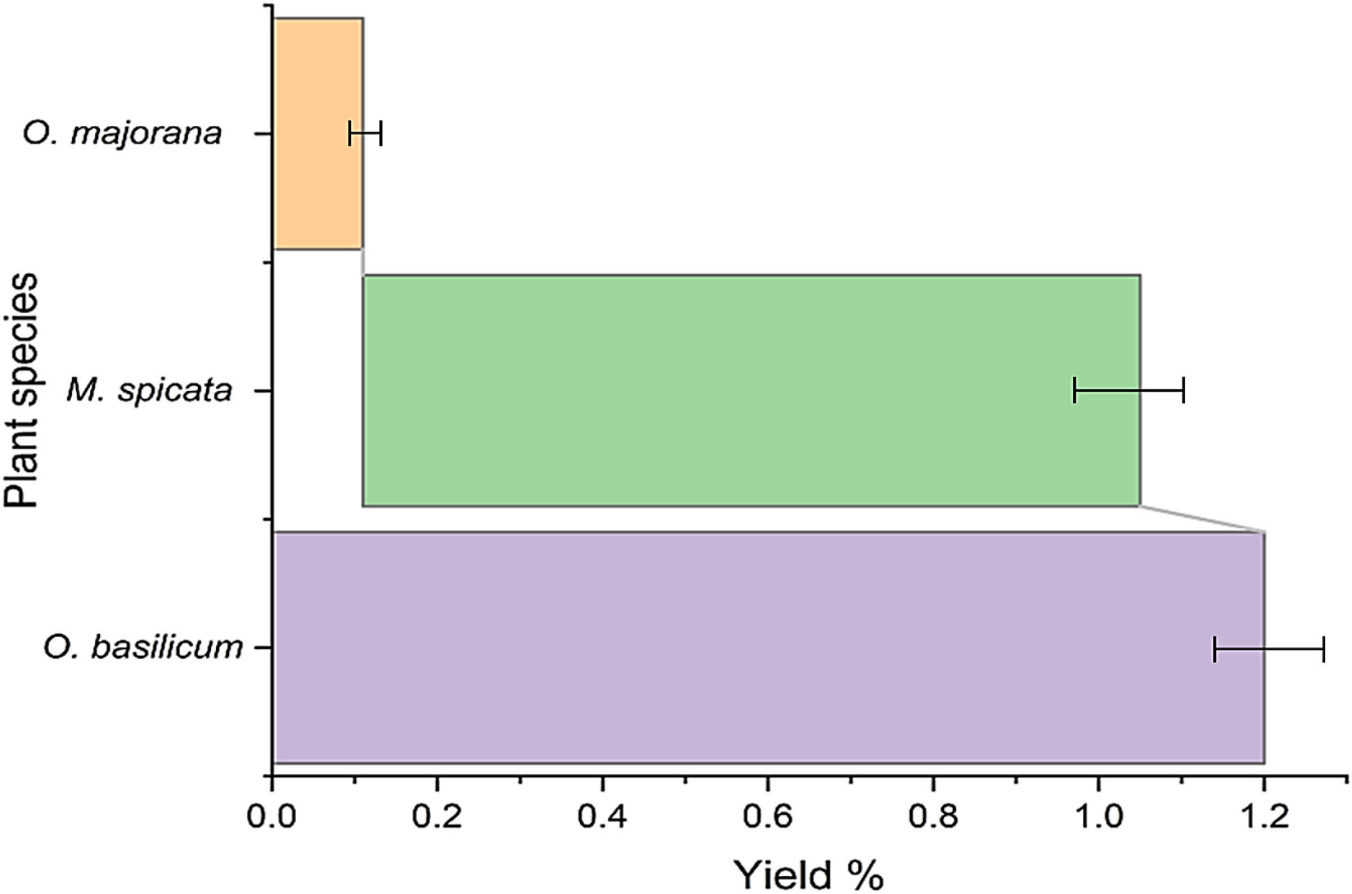
Yield percentages of *O*. *majorana*, *M.* sp*icata* and *O*. *basilicum* oils extraction.

The EO yields obtained from the leaves of the plants *O. majorana*, *M.* sp*icata* and *O. basilicum* using the hydro-distillation method were 0.11, 0.94 and 1.20% (**Figure 1**), respectively. These percentages represent the amount of essential oil extracted relative to the weight of the plant material used. It is important to note that the extraction yield can vary depending on the extraction technique used and the organic solvent choice, which is highlighted in the study by Pavela et al (Pavela et al., 2010). Hydro-distillation is one of the common methods used for EO extraction, but other methods and solvents could result in different yields.

### 3.2 Chemical composition analysis via GC-MS

To provide a comprehensive understanding of the chemical profiles of EOs derived from different species, detailed analysis via GC-mass spectrometry (GC-MS) revealed significant constituents across *O. majorana*, *M.* sp*icata* and *B O. basilicum*. The GC-MS analysis of *O. majorana* identified a total of 47 compounds, with terpinen-4-ol being the predominant compound at 25.47% (0.2547 ± 0.0127), followed by sabinene at 18.41% (0.1841 ± 0.0055). In contrast, *M.* sp*icata* comprised 37 compounds, with piperitenone oxide at 43.83% (0.4383 ± 0.0219) as the most abundant, complemented by 3,6,6-trimethyl-cyclohex-2-enone at 12.98% (0.1298 ± 0.0065). Meanwhile, the essential oil of *O. basilicum* contained 27 compounds, featuring Methyl (E)-cinnamate at 48.69% (0.4869 ± 0.0243) and Camphor at 16.37% (0.1637 ± 0.0082) as its dominant constituents (**Supplementary File 1**, **Supplementary Table S1**).

Additionally, the presence of linalool in the essential oil of *O. basilicum* at 5.13% (0.0513 ± 0.0026) stands out, as linalool is known for its pleasant aroma and potential therapeutic properties, including anti-inflammatory and anxiolytic effects.

The GC-MS profiles (**Supplementary File 2, Supplementary Figures S1**-**Supplementary File 2, Supplementary Figures S3**) showcase distinct chromatograms corresponding to the unique volatile compounds present in each essential oil, with detailed composition provided in **Supplementary Table S1**.

### 3.3 Larvicidal activity

The toxicity of the essential oils was evaluated by probit analysis, with the hexane control included as the baseline (0% concentration) for each oil. The toxicity of EOs was evaluated by probit analysis of corrected mortality data, with the hexane control included as the baseline (0% concentration) for each oil. The EOs extracted from *O. majorana*, *M.* sp*icata* and *O. basilicum* leaves demonstrated toxicity towards the 3^rd^ instar larvae of *S. littoralis*. Among these oils, *M.* sp*icata* exhibited the highest efficacy against the larvae, with an LC_50_ value of 0.43% (fiducial limits: 0.32-0.52). This was followed by *O. basilicum* with an LC_50_ of 0.51% (fiducial limits: 0.42-0.60) and *O. majorana* with an LC_50_ of 1.18% (fiducial limits: 0.73-1.72). The synthetic positive control, lambda-cyhalothrin, showed the highest toxicity with an LC_50_ of 0.007% (fiducial limits: 0.004-0.011) (**Table 2**).

**Table 2:** Toxicity of essential oils from *O. majorana*, *M.* sp*icata*, and *O. basilicum* against third instar larvae of *S. littoralis* after a 3-hour topical exposure (Probit analysis includes control as 0% concentration).

Species	LC_50_ (95% FL)	Slope (± SEM)	*Chi-square (X²)*	*df*	*Statistical Comparison of LC_50_¹*
O. majorana	1.18 (0.73-1.72)	2.95 ± 0.48	~1.8	3	a
*M.* sp*icata*	0.43 (0.32-0.52)	2.95 ± 0.25	~1.1	4	b
*O. basilicum*	0.51 (0.42-0.60)	4.10 ± 0.50	~2.5	6	b
*Lambda-cyhalothrin*	0.007 (0.004-0.011)	3.25 ± 0.52	~1.2	3	c

LC, lethal concentration; FL, fiducial limits; SEM, standard error of the mean; df, degrees of freedom. ¹ Mortality data were corrected using Abbott’s formula (Abbott, 1925) where applicable. The hexane-only control was included as the 0% concentration in the probit regression model for each essential oil. LC_50_ values followed by different letters (a, b, c) are significantly different based on a test for equality and parallelism of probit regression lines.

The mortality of 3^rd^-instar *S. littoralis* larvae was assessed at 24, 48, 72, and 96 hrs after topical exposure to essential oils of *O. majorana* (0.5-4.0%), *M.* sp*icata* (0.25-1.5%), *O. basilicum* (0.3–1.5%), and the positive control lambda-cyhalothrin (0.001-0.02%) (**Table 3**).

**Table 3:** Mean cumulative percentage mortality (± SEM) of third-instar *S. littoralis* larvae over time following a 3-hour topical exposure to hexane-only control and different concentrations of *O. majorana*, *M.* sp*icata*, *O. basilicum* essential oils, and lambda-cyhalothrin.

Treatment	Concentration (% w/v)	Mortality at 24 h (%)	Mortality at 48 h (%)	Mortality at 72 h (%)	Mortality at 96 h (%)
Control	0 (Hexane-only)	0.0 ± 0.0	0.0 ± 0.0	0.0 ± 0.0	3.3 ± 1.7
*Origanum majorana*	0.5	6.7 ± 1.7 ^c^	13.3 ± 1.7 ^d^	20.0 ± 0.0 ^d^	26.7 ± 1.7 ^d^
	1	10.0 ± 0.0 ^c^	20.0 ± 0.0 ^c^	30.0 ± 0.0 ^c^	36.7 ± 1.7 ^c^
	2	23.3 ± 1.7 ^b^	36.7 ± 1.7 ^b^	43.3 ± 1.7 ^b^	50.0 ± 0.0 ^b^
	3	36.7 ± 1.7 ^a^	50.0 ± 0.0 ^a^	56.7 ± 1.7 ^a^	63.3 ± 1.7 ^a^
	4	40.0 ± 0.0 ^a^	53.3 ± 1.7 ^a^	60.0 ± 0.0 ^a^	66.7 ± 1.7 ^a^
*Mentha*	0.25	10.0 ± 0.0 ^d^	20.0 ± 0.0 ^e^	26.7 ± 1.7 ^e^	33.3 ± 1.7 ^e^
	0.5	20.0 ± 0.0 ^c^	33.3 ± 1.7 ^d^	40.0 ± 0.0 ^d^	46.7 ± 1.7 ^d^
	0.75	43.3 ± 1.7 ^b^	56.7 ± 1.7 ^b^	63.3 ± 1.7 ^b^	70.0 ± 0.0 ^b^
	1.5	56.7 ± 1.7 ^a^	70.0 ± 0.0 ^a^	76.7 ± 1.7 ^a^	83.3 ± 1.7 ^a^
*Ocimum basilicum*	0.3	13.3 ± 1.7 ^d^	23.3 ± 1.7 ^d^	30.0 ± 0.0 ^d^	36.7 ± 1.7 ^d^
	0.7	43.3 ± 1.7 ^c^	56.7 ± 1.7 ^c^	63.3 ± 1.7 ^c^	70.0 ± 0.0 ^c^
	1	63.3 ± 1.7 ^a^	76.7 ± 1.7 ^a^	83.3 ± 1.7 ^a^	90.0 ± 0.0 ^a^
	1.5	66.7 ± 1.7 ^a^	80.0 ± 0.0 ^a^	86.7 ± 1.7 ^a^	93.3 ± 1.7 ^a^
*Lambda-cyhalothrin*	0.001	36.7 ± 3.3 ^d^	60.0 ± 0.0 ^d^	76.7 ± 3.3 ^c^	83.3 ± 3.3 ^c^
	0.005	73.3 ± 3.3 ^b^	90.0 ± 0.0 ^b^	96.7 ± 3.3 ^b^	100.0 ± 0.0 ^b^
	0.01	96.7 ± 3.3 ^a^	100.0 ± 0.0 ^a^	100.0 ± 0.0 ^a^	100.0 ± 0.0 ^a^
	0.02	100.0 ± 0.0 ^a^	100.0 ± 0.0 ^a^	100.0 ± 0.0 ^a^	100.0 ± 0.0 ^a^

*Data are presented as mean observed (raw) mortality percentage ± SEM (n=3 replicates of 10 larvae each). For each treatment (essential oil or insecticide), mean percentages at each time point followed by different lowercase superscript letters (a–e) are significantly different from each other (One-way ANOVA, Tukey’s HSD test, *p* <no><</no> 0.05). All treatment concentrations were statistically compared against the single hexane-only control group. Note: The Abbott-corrected mortality values (calculated as per Section 2.7) were used for the probit analysis in **Table 2**, while this table presents the raw observational data for clarity across time points.3.4 Molecular determination of the larval defense system against oils treatment

Mortality increased in a clear concentration- and time-dependent manner for all three oils. At 96 hrs, mortality for *O. majorana* ranged from 26.7% (at 0.5%) to 66.7% (at 4.0%). *M.* sp*icata* induced 33.3 to 86.7% mortality, and *O. basilicum* was the most potent, with mortality ranging from 36.7 to 93.3%. Statistical analysis confirmed significant differences between concentrations at each time point (*p* < 0.05). Mortality in the hexane control group was negligible (≤ 3.3%).

The expression of three key defense-related genes (*PR1*, *PR2* and *Chitinase*) was analyzed in *S. littoralis* larvae 24 hrs after topical exposure to hexane-only (control) or sub-lethal concentrations of *O. basilicum* or *M.* sp*icata* essential oils. Gene expression levels in EO-treated larvae are expressed as fold-change relative to the hexane-only control group.

**Figure 2:**
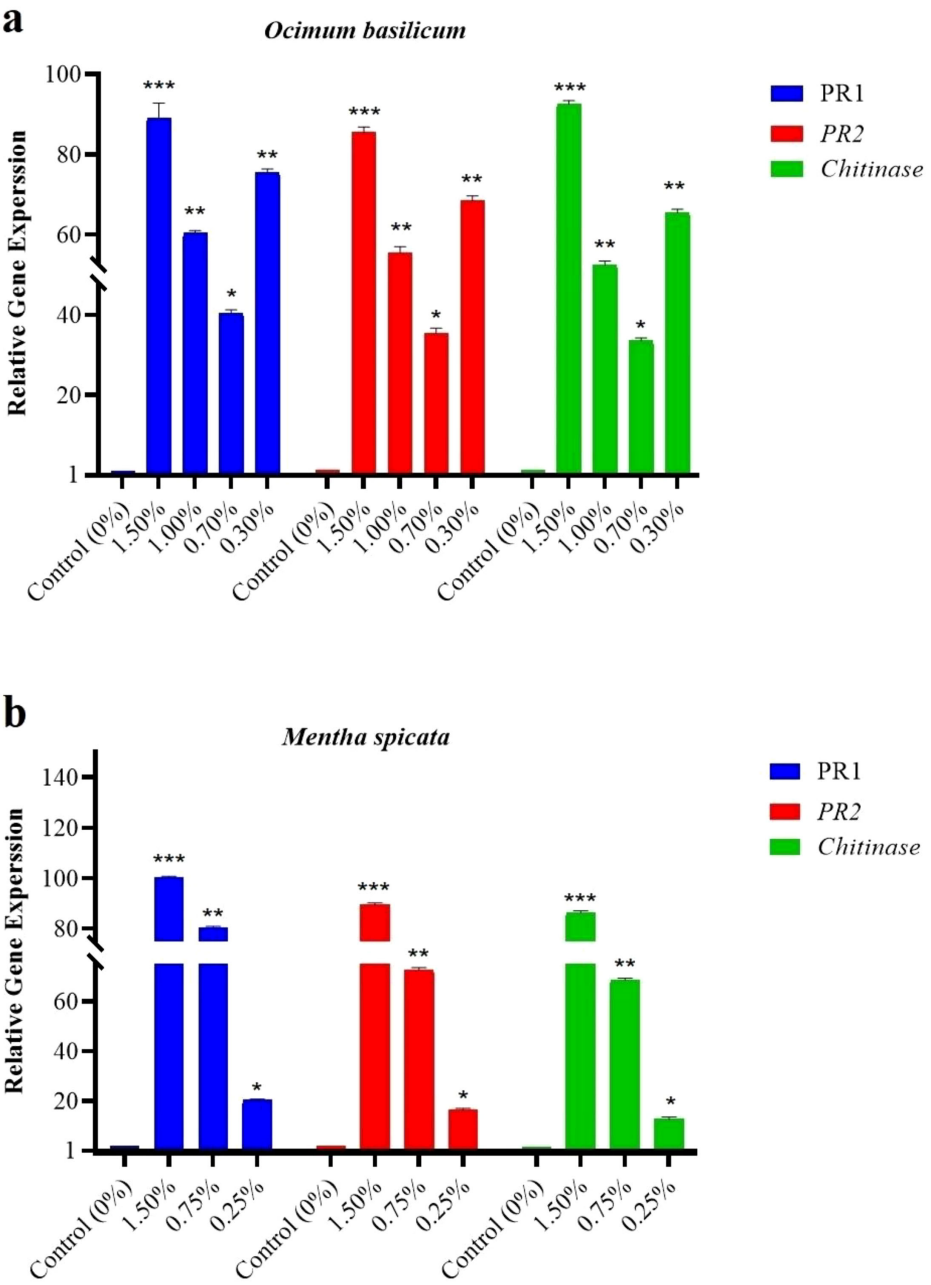
Expression of defense-related genes in *S. littoralis* larvae 24 hrs after essential oil exposure. Third-instar larvae were topically treated with hexane-only (0%, control) or sub-lethal concentrations of *O. basilicum* (0.3, 0.7, 1.0 and 1.5%) or *M.* sp*icata* (0.25, 0.75 and 1.5%) essential oil. Relative expression of *PR1*, *PR2* and *Chitinase* was determined by qRT-PCR, normalized to the *18S rRNA* housekeeping gene, and calculated using the 2^-ΔΔCT^ method relative to the hexane-only control (baseline set at 1). All EO treatments are expressed as fold-change relative to this solvent control (dashed line). Data represent the mean ± SEM of three biological replicates. Asterisks denote significant differences from the control (*p < 0.05, **p < 0.01, ***p < 0.001).

Treatment with *O. basilicum* EO induced a potent and concentration-dependent upregulation of all target genes. At the highest concentration tested (1.5%), *PR1, PR2*, and *Chitinase* expression increased dramatically to approximately 85-fold, 85-fold, and 93-fold of control levels, respectively (*p* < 0.001). This strong induction displayed a clear dose-response, with lower concentrations (1.0%, 0.7% and 0.3%) eliciting proportionally significant yet reduced upregulation (**Figure 2A**).

In contrast, *M.* sp*icata* EO elicited a more varied transcriptional response. While the 1.5% concentration also caused significant upregulation across all genes (reaching ~100-fold for *PR1*), the effect at lower concentrations was less uniform. Notably, at the 0.25% concentration, the fold-change increase was markedly lower (e.g., ~20-fold for *PR1*, ~12-fold for *Chitinase*), suggesting a steeper dose-response gradient compared to *O. basilicum* (**Figure 2B**). For both essential oils, all treatments resulted in statistically significant differences from the hexane-treated control group (*p* < 0.05).

### 3.5 Differential display PCR

**Figure 3:**
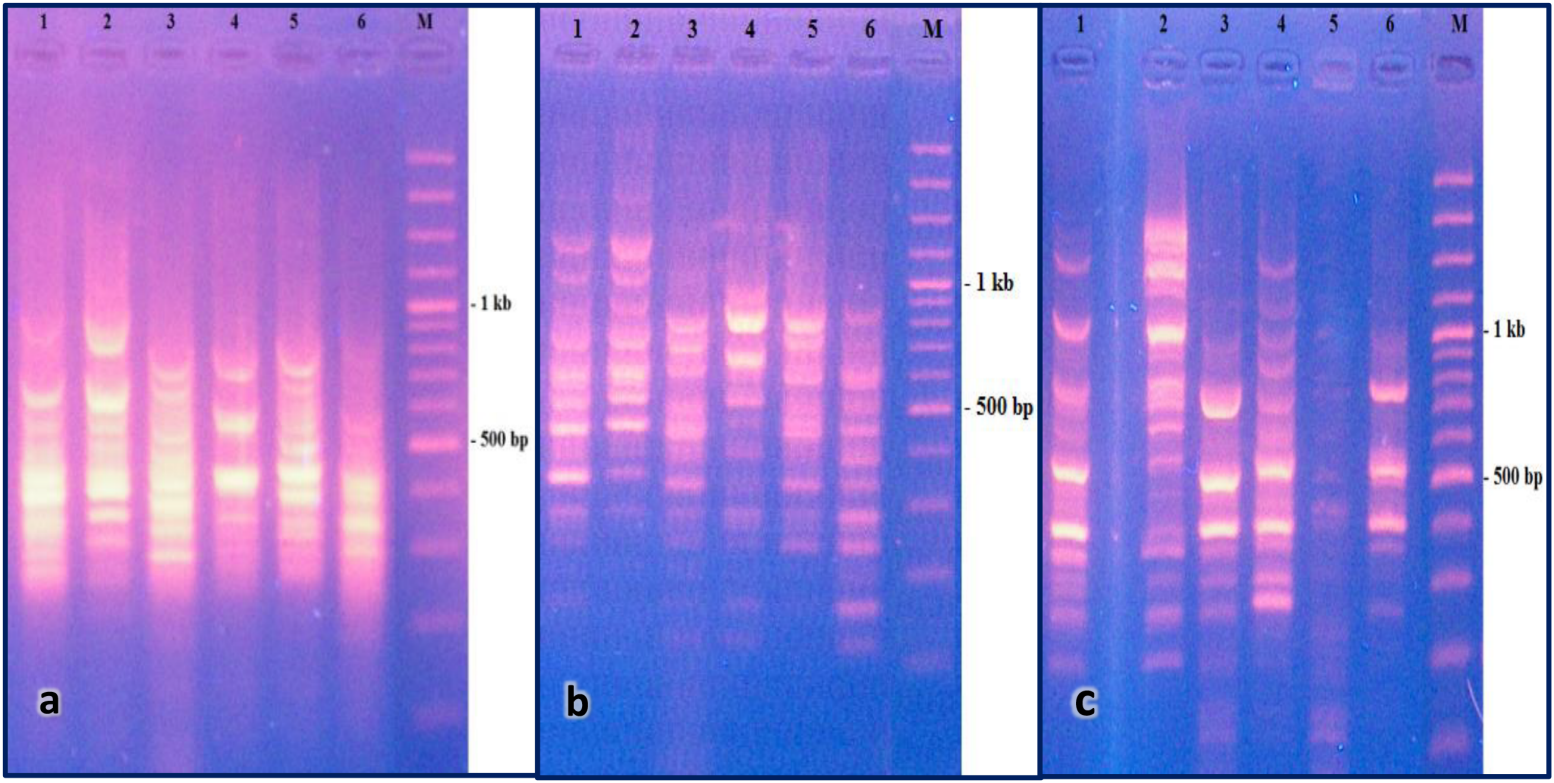
Gel electrophoresis pattern from differential display PCR using **(a)***PR1*, **(b)***PR2*, and **(c)***Chitinase* primers. M: DNA Marker. Lanes 1 and 2: cDNA from surviving larvae after treatment with 1.5% and 0.7% *O. basilicum* oil, respectively. Lane 3: untreated control (hexane-only). Lanes 4, 5, and 6: cDNA from larvae that died after treatment with 1%, 0.7%, and 0.3% *O. basilicum* oil, respectively.

Genetic variations between the control and treated cotton leafworms were investigated. Notably, with *O. basilicum*, the three primers exhibited distinct band patterns in treated versus untreated insects (**Figure 3** and **Supplementary Figures S4**-**S6**). Primer *PR1* generated approximately 43 bands ranging in molecular size from 1k to 100bp, while *PR2* yielded 53 bands with sizes ranging from 3k to 50 bp. Similarly, the chitinase primer produced 56 bands of various molecular sizes spanning from 3k to 50 bp.

Post-treatment, *PR1* and *PR2* in surviving worms exposed to *O. basilicum* displayed up-regulated bands at approximately 850 and 820 bp respectively, whereas bands at around 520 and 1300 bp were induced with the chitinase primer. Conversely, deceased worms following *O. basilicum* treatment exhibited down-regulated bands at approximately 550 and 600 bp with *PR1* and *PR2*, respectively.

**Figure 4:**
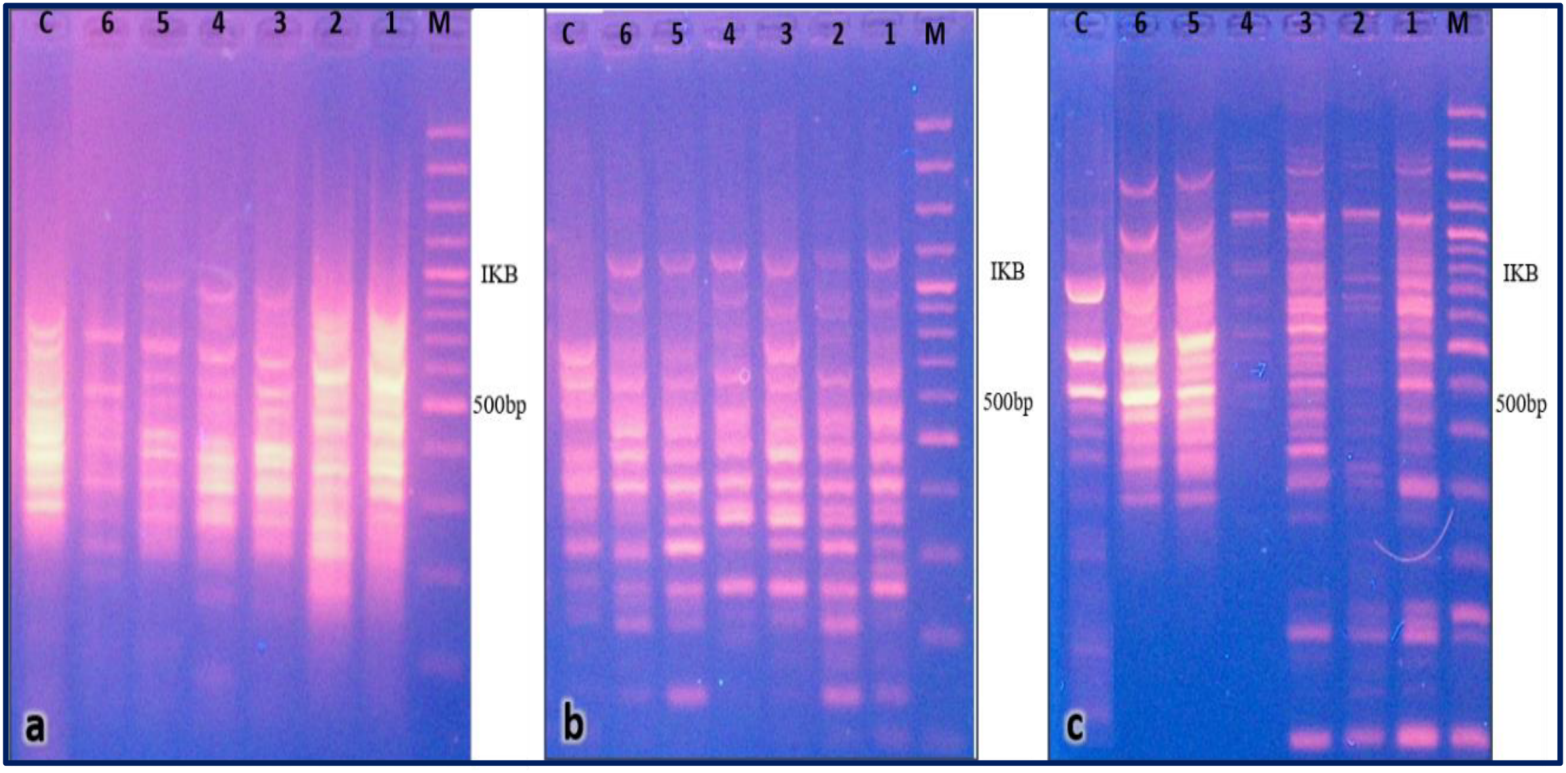
Gel electrophoresis pattern from differential display PCR using **(a)***PR1*, **(b)***PR2*, and **(c)***Chitinase* primers. M: DNA Marker. Lanes 1 and 2: cDNA from surviving larvae after treatment with 1.5% and 0.7% *M.* sp*icata* oil, respectively. Lane 3: untreated control (hexane-only). Lanes 4, 5, and 6: cDNA from larvae that died after treatment with 1%, 0.7%, and 0.3% *M.* sp*icata* oil, respectively.

In the case of *M.* sp*icata*, *PR1* primers successfully amplified approximately 56 bands with diverse molecular sizes ranging from 1k to 100bp. Conversely, *PR2* enabled the amplification of 79 bands, with molecular sizes ranging from 2k to 50bp. Additionally, the Chitinase primer amplified around 83 bands with molecular sizes ranging from 3k to 50bp. Most of the bands obtained were monomorphic, while the polymorphic bands were categorized into up-regulated and down-regulated bands. Up-regulated bands were detected at approximately 850 and 650 bp, whereas down-regulated bands were seen at 600 bp when utilizing *PR1* (**Figure 4** and **Supplementary Figures S7**-**S9**). For *PR2*, up-regulated bands appeared at around 480 bp and down-regulated bands at around 520 bp. Notably, a distinctive up-regulation band was identified at approximately 550 bp using the Chitinase primer. The up-regulation of immune genes in surviving *S. littoralis* larvae indicates the insect’s resistance to the effects of the oils.

## 4 Discussion

### 4.1 Chemical composition and insecticidal potential of Lamiaceae essential oils

The production of complex mixtures of volatile terpenes and phenylpropanoids is a hallmark of the Lamiaceae family, serving as a core chemical defense strategy against herbivores and pathogens (Inanoglu et al., 2023; Kowalczyk et al., 2023). The insecticidal activity of these EOs is not merely a function of their complexity but is driven by the specific modes of action of their dominant constituents. In our study, the efficacy of *O. majorana* EO can be attributed to its high concentrations of terpinen-4-ol (25.47%) and sabinene (18.41%). Terpinen-4-ol, a monoterpene alcohol, is known to exhibit neurotoxic effects in insects, including the inhibition of AChE and disruption of octopaminergic signaling, leading to paralysis and death (Pérez-López et al., 2006; Pavela et al., 2010; Sales et al, 2020; Chigo-Hernandez and Tomasino, 2023). Sabinene, a bicyclic monoterpene, contributes through its action as a potent feeding deterrent and by compromising cuticular and cellular membranes, facilitating the penetration of other toxicants (Chigo-Hernandez and Tomasino, 2023).

The insecticidal potential of plant essential oils is influenced by multiple factors, beginning with their production. Abiotic conditions and cultivation practices can significantly modulate essential oil yield and chemical profile, underscoring the importance of standardized plant material for reproducible bioactivity (Kaur et al., 2017). Within this framework, the Lamiaceae family stands as a preeminent source of insecticidal volatiles, with diverse species from various regions demonstrating potent effects against a range of arthropod pests (Mamadalieva et al., 2017). Research also confirms that significant bioactivity extends beyond Lamiaceae to other families such as Zingiberaceae and Rutaceae, broadening the resource base for discovery (Visakh et al., 2022; Anuranj et al., 2024). Crucially, the efficacy of an essential oil is predominantly governed by the mode of action of its major constituents, which dictate its biological activity (Yang et al., 2023). This activity can be deployed through various application methods, from the contact toxicity central to this study to established fumigant or repellent actions against other pests, highlighting their practical versatility (El-Seedi et al., 2014; Moura et al., 2024).

Similarly, the high toxicity of *M.* sp*icata* EO correlates with its major component, piperitenone oxide (43.83%). This ketone is recognized for its strong fumigant and contact toxicity, which is linked to its ability to interfere with neuromodulation and energy metabolism in insects (Kakouri et al., 2022; Prabhakar et al., 2024). This aligns with broader evidence within the *Mentha* genus, where other ketones like carvone have been identified as primary agents conferring strong insecticidal properties to essential oils (Yang et al., 2023). For *O. basilicum*, the dominant methyl (E)-cinnamate (48.69%) is a key insecticidal agent. Its activity is associated with both neurotoxicity and the inhibition of critical detoxification enzymes like esterases, enhancing its potency against lepidopteran larvae (Rodríguez-González et al., 2019).

The chemical structure of these compounds is directly relevant to their bioactivity; for instance, the presence of functional groups like the hydroxyl group in terpinen-4-ol or the α,β-unsaturated ester in methyl cinnamate are often involved in interactions with target enzymes or receptors (Pérez-López et al., 2006; Chigo-Hernandez and Tomasino, 2023). From an ecological perspective, these compounds are synthesized by the plant as direct defenses, deterring herbivory, reducing oviposition, and impairing larval development. Their insecticidal activity in our bioassays thus reflects the successful exploitation of the plant’s own evolved defense chemistry for pest management.

### 4.2 Larvicidal efficacy and concentration-response relationships

The toxicity of the essential oils exhibited a clear dose- and time-dependent relationship. While the highest concentrations induced substantial mortality within 24 hrs, a progressive accumulation of lethal effects was observed at lower, sublethal doses over the full 96-hour observation period (**Figure 2**). This underscores that the overall insecticidal efficacy is a function of both concentration and exposure duration, a critical parameter for field application. Furthermore, the variation in potency among the oils, as reflected in their LC_50_ values, suggests that bioactivity arises not from single compounds in isolation but from the integrated effect of the complete chemical profile, where synergistic interactions likely play a key role.

To contextualize the larvicidal potency of these botanical extracts, their efficacy was benchmarked against a conventional synthetic insecticide used as a positive control. The inclusion of a synthetic pyrethroid, lambda-cyhalothrin, as a positive control provided a critical benchmark for efficacy. Its LC_50_ (0.007%) was significantly lower than those of the tested essential oils, confirming the superior acute toxicity of this conventional insecticide. This expected result highlights the different roles these agents can play in IPM. While synthetic insecticides offer high immediate potency, plant-derived essential oils provide a complex, multi-mechanistic alternative. Their substantial toxicity within a 1-2% application range, coupled with their potential for reduced environmental persistence and different resistance mechanisms, supports their use as complementary tools in IPM strategies, particularly where the mitigation of chemical residues and resistance development is prioritized.

This demonstrated potential for effective control (Kocher et al., 2024) necessitates a holistic safety assessment, including an investigation of the sublethal impacts on insect physiology, which can reveal novel modes of action beyond direct mortality (Castilhos et al, 2018).

### 4.3 Modulation of larval immune response and genetic regulation

The essential oils of *O. basilicum* and *M.* sp*icata* induced a profound molecular response in *S. littoralis* larvae, indicating a novel sublethal mode of action that extends beyond acute toxicity. qRT-PCR analysis revealed a potent, dose-dependent upregulation of key defense-related genes (*PR1*, *PR2*, and *Chitinase*), with expression levels reaching up to approximately 100-fold that of the control in some treatments (**Figure 2**).

This massive transcriptional induction suggests a state of extreme immunological and physiological stress triggered by essential oil exposure (Krishnan and Kodrík, 2006). The strong upregulation of defensin genes (*PR1* and *PR2*) points to a systemic activation of humoral immune pathways, likely an attempt to counter perceived microbial challenge or tissue damage (Siddiqui et al., 2023). The significant induction of *Chitinase*, an enzyme critical for chitin remodeling, may indicate a compensatory mechanism to repair EO-induced damage to the chitinous cuticle or peritrophic matrix, which are vital barriers against pathogens and environmental stress (Krishnan and Kodrík, 2006; Nasr et al, 2010).

Notably, the differential potency and dose-response patterns between the two oils with *O. basilicum* showing a more graded effect across concentrations may be linked to their distinct major chemical constituents (methyl cinnamate *vs.* piperitenone oxide) and their specific interactions with insect cellular targets. This dysregulation of fundamental defense and structural genes likely contributes to reduced larval fitness, impairing their ability to mount an effective immune response and potentially increasing susceptibility to secondary stressors.

The differential display PCR analysis provided further genetic evidence of this immune activation, revealing distinct banding patterns between treated and control insects (**Figures 3**, **4**). Specific up-regulated bands in surviving larvae such as fragments at approximately 850 bp with *PR1* corroborate the enhanced expression of immune-related transcripts detected by qRT-PCR. Conversely, down-regulated bands in deceased larvae suggest a collapse of normal regulatory processes under lethal EO concentrations. This genetic evidence aligns with studies on other lepidopterans, where plant secondary metabolites have been shown to trigger defensive gene expression as part of a generalized stress response (Nasr et al, 2010). Therefore, the sublethal action of these EOs imposes a dual burden: direct toxic effects coupled with the metabolic cost of mounting an immune defense, which may collectively impair larval development, fecundity, and overall fitness, adding a valuable dimension to their mode of action in IPM.

### 4.4 Broader bioactivity and ecological implications

Building on the observed larvicidal and immunomodulatory effects, the insecticidal activity of these essential oils may extend to a wider spectrum of arthropod pests, underscoring their potential as multi-target botanical agents. For instance, the high toxicity of a methyl cinnamate-rich *O. basilicum* chemotype against the spider mite *Tetranychus urticae* (Moura et al., 2024) provides complementary evidence for the broad bioactivity of this key phenylpropanoid. This correlation suggests that methyl (E)-cinnamate, the dominant compound responsible for larvicidal activity in our study, may also be a primary agent in its acaricidal effects, highlighting a common chemical mechanism for cross-pest efficacy (Rodríguez-González et al., 2019; Worku et al., 2024). Similarly, the established repellent and toxic properties of *M.* sp*icata* and *O. majorana* EOs against other pests, such as ticks, further support the value of these plant-derived compounds in integrated vector management (El-Seedi et al., 2014).

From an ecological perspective, the use of such EOs aligns with the principles of sustainable agriculture. Their natural origin typically facilitates faster environmental degradation compared to persistent synthetic insecticides, potentially reducing long-term ecological residue and pollution (Pavela, 2015). However, this broader activity necessitates a critical consideration of non-target effects. As highlighted by Pavela et al (Pavela, 2015). and Kocher et al (Kocher et al., 2024), even plant-derived insecticides can impact beneficial arthropods, including pollinators and natural predators. Therefore, the “eco-friendly” label must be applied cautiously, contingent on targeted application and selectivity studies.

For practical implementation, the volatility and photosensitivity of EOs pose significant challenges for field persistence. Future formulations, such as nanoemulsions or encapsulation in polymer matrices, will be crucial to enhance their stability, prolong their bioavailability on crops, and potentially mitigate negative impacts on non-target organisms (Pavela, 2015; Prabhakar et al., 2024). Consequently, while this study confirms the strong *in vitro* potential of *O. majorana*, *M.* sp*icata*, and *O. basilicum* EOs, their successful translation into reliable agricultural tools depends on overcoming these formulation hurdles and validating efficacy through controlled field trials.

Briefly, while the present study underscores the promising insecticidal properties of these EOs, further explorations are warranted to address their long-term effects, potential phytotoxicity to non-target organisms and optimization of application methods. By broadening the research scope, the practical applications of these EOs in sustainable pest management strategies can be fully realized, paving the way for safer agricultural practices.

## 5 Conclusions

This study demonstrates that the distinct insecticidal properties of essential oils from *O. majorana*, *M.* sp*icata*, and *O. basilicum* against *S. littoralis* larvae correspond to their unique phytochemical profiles. The toxicity of *O. majorana* correlates with a profile rich in terpinen-4-ol and sabinene, while the superior efficacy of *M.* sp*icata* and *O. basilicum* correlates with oils dominated by piperitenone oxide and methyl (E)-cinnamate, respectively. It is important to note that, as complex mixtures, the observed bioactivity likely results from synergistic interactions among the various constituents rather than the action of single compounds in isolation. Furthermore, these EOs, particularly from *M.* sp*icata* and *O. basilicum*, elicited a significant upregulation of defense-related genes (*PR1*, *PR2*, and *Chitinase*), indicating that sublethal exposure triggers a substantial immune response, adding a physiological stress component to their mode of action. These findings have significant environmental and agricultural implications. The potent insecticidal and gene-modulating effects position these EOs as promising candidates for eco-friendly pest management strategies. Incorporating such bio-based solutions can reduce reliance on synthetic chemical pesticides, helping to mitigate environmental impact and promote agricultural sustainability.

## Data Availability

The datasets presented in this study can be found in online repositories. The names of the repository/repositories and accession number(s) can be found in the article/**Supplementary Material**.
