# Supplementary material for: Chemical composition of Origanum majorana, Mentha spicata and Ocimum basilicum essential oils and their impact on Spodopteralittoralis: toxicity and immune response
Source: Front Plant Sci. 2026 Jan 12;16:1737742. doi: 10.3389/fpls.2025.1737742 (PMC12832627; doi:10.3389/fpls.2025.1737742)
Supplement: Supplementary file 1 [file DataSheet1.pdf]

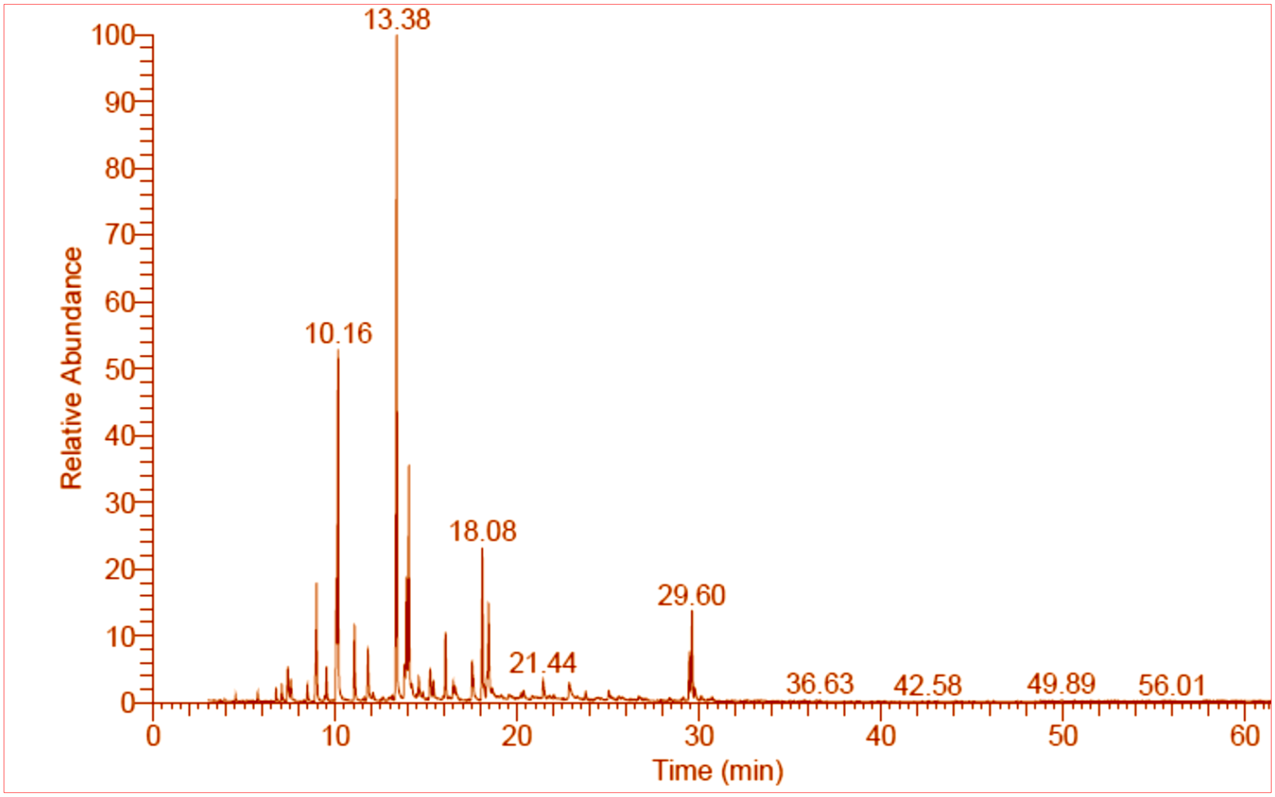

Figure S1. Chromatogram of *O. majorana* essential oil

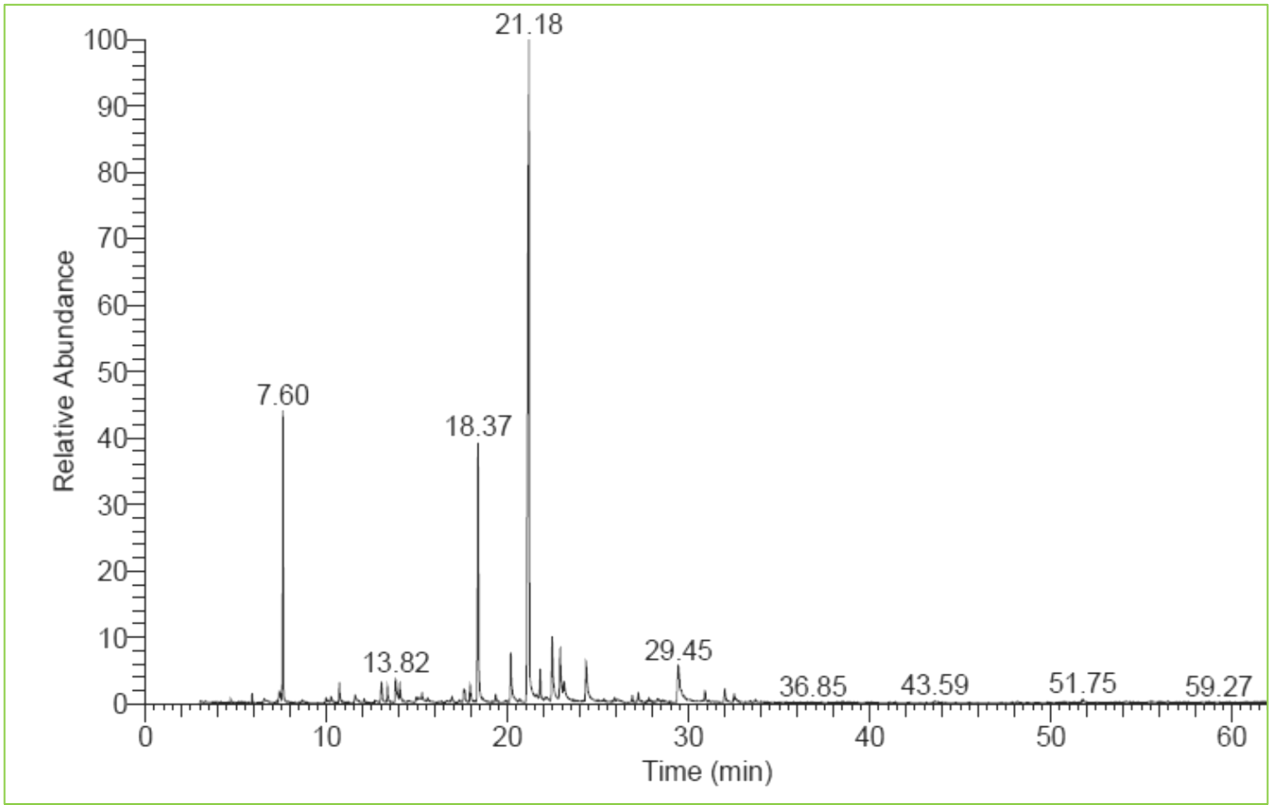

Figure S2. Chromatogram of *M. spicata* essential oil

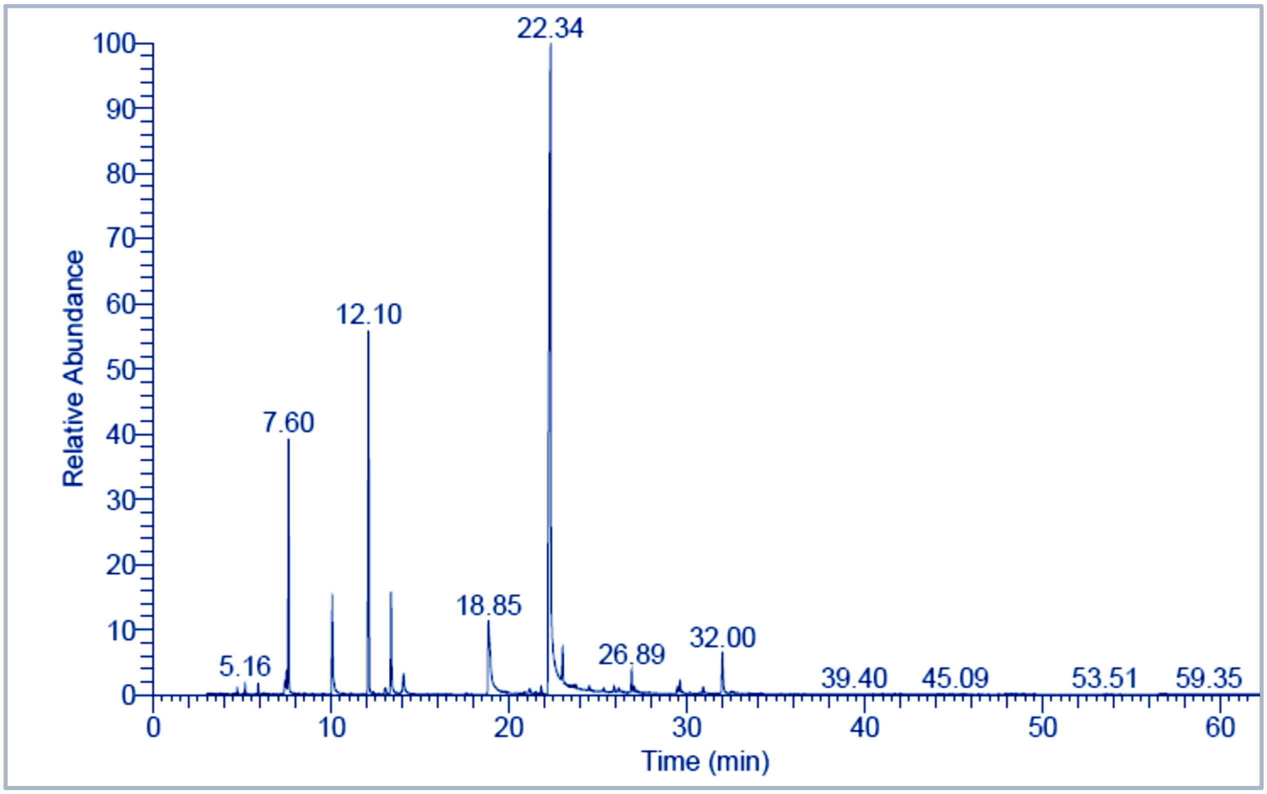

Figure S3. Chromatogram of *O. basilicum* essential oil

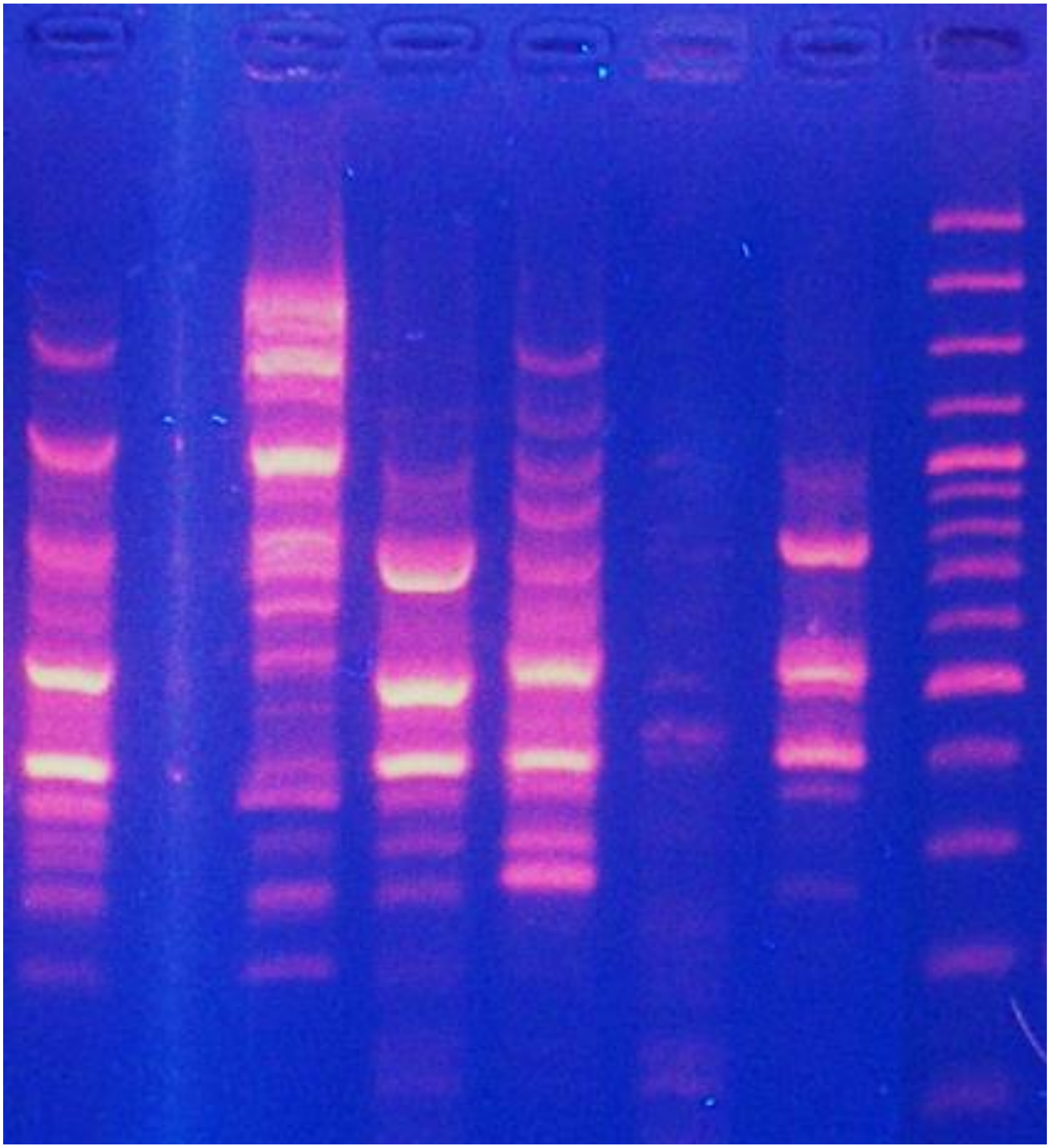

Figure S4. Gel electrophoresis pattern using; *Chitenase* primer; **M**: DNA Marker, **lanes 1** and **2**: alive worms after the treatment with 1.5 and 0.7% concentrations of *O. basilicum* oil, respectively. **Lane 3**: control, **lanes 4**, **5** and **6**: dead worms after the treatment with 1, 0.7 and 0.3% concentrations of *O. basilicum* oil, respectively.

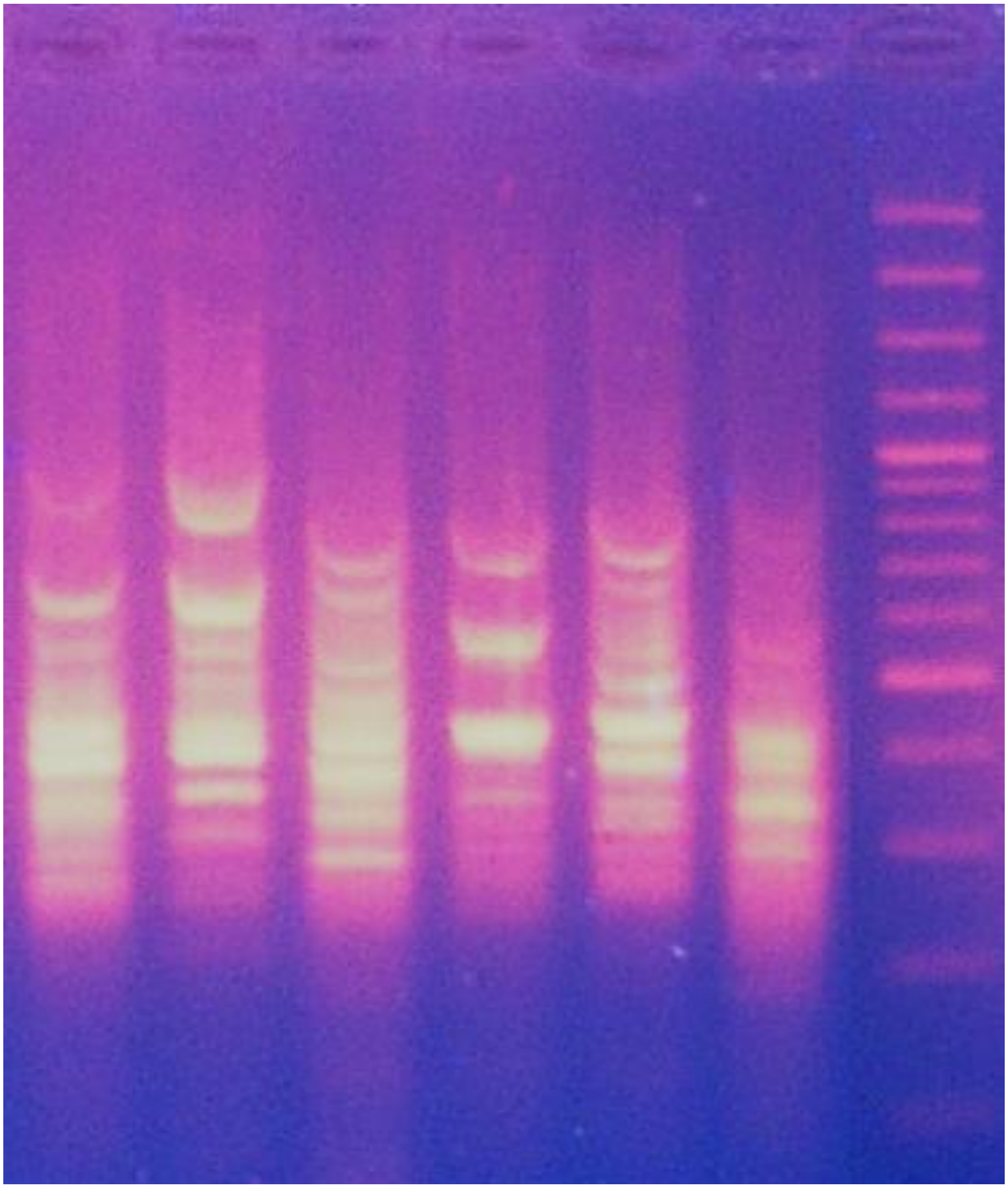

Figure S5. Gel electrophoresis pattern using; *PR1* primer; **M**: DNA Marker, **lanes 1** and **2**: alive worms after the treatment with 1.5 and 0.7% concentrations of *O. basilicum* oil, respectively. **Lane 3**: control, **lanes 4**, **5** and **6**: dead worms after the treatment with 1, 0.7 and 0.3% concentrations of *O. basilicum* oil, respectively.

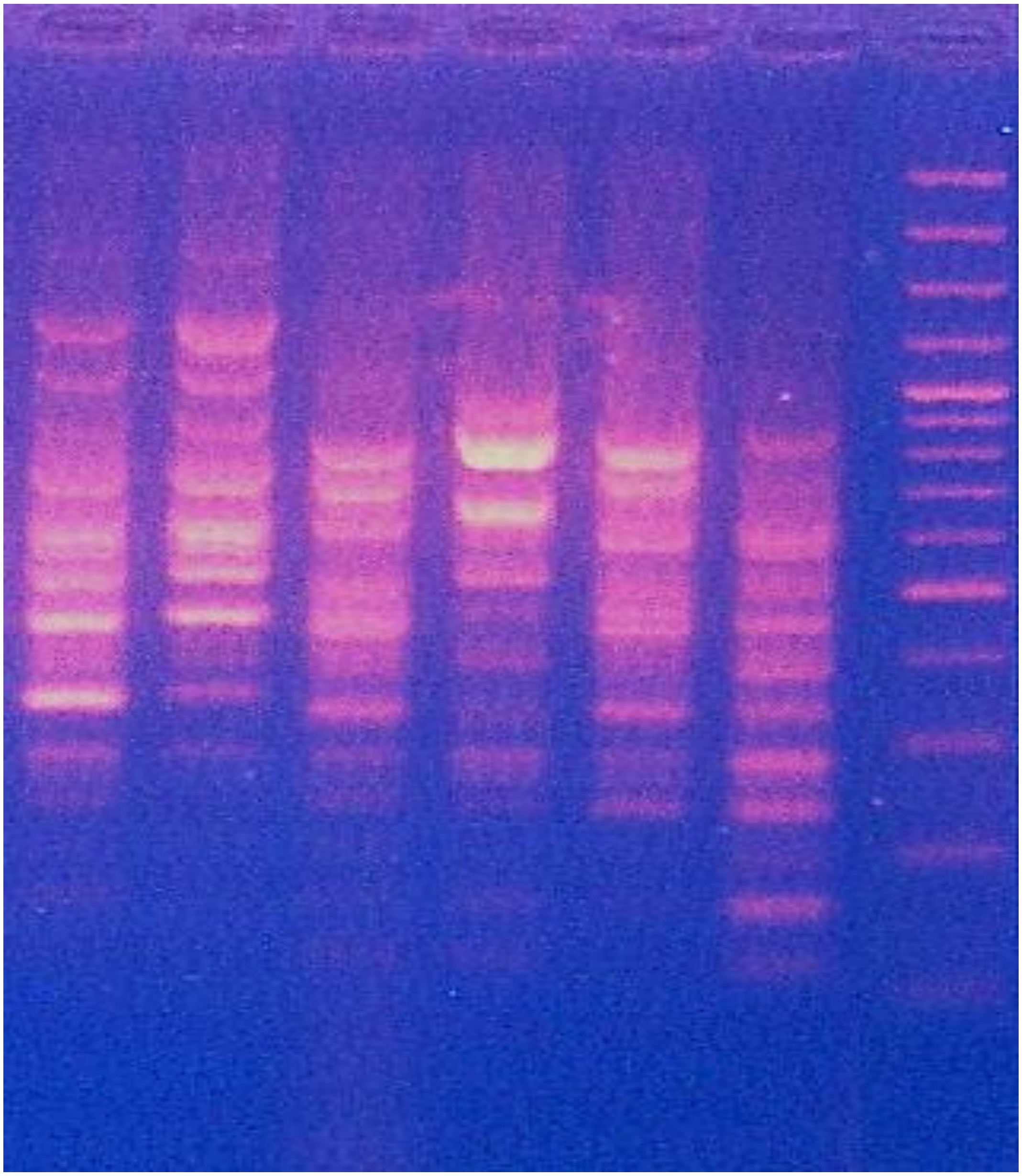

Figure S6. Gel electrophoresis pattern using; *PR2* gene; **M**: DNA Marker, **lanes 1** and **2**: alive worms after the treatment with 1.5 and 0.7% concentrations of *O. basilicum* oil, respectively. **Lane 3**: control, **lanes 4**, **5** and **6**: dead worms after the treatment with 1, 0.7 and 0.3% concentrations of *O. basilicum* oil, respectively.

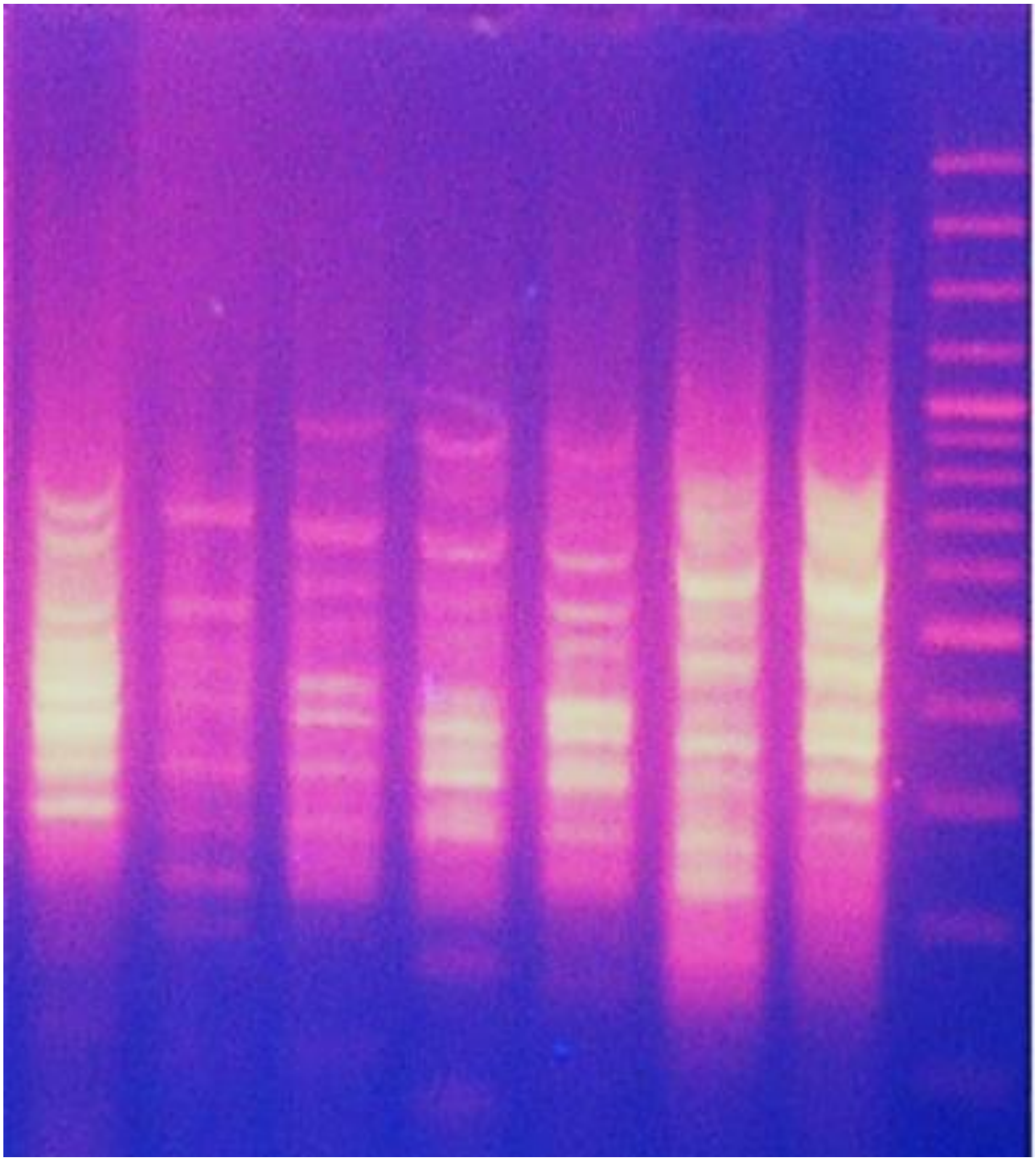

Figure S7. Gel electrophoresis pattern using *PR1* primer. **M**: DNA Marker, **lanes 1** and **2**: alive worms after the treatment with 1.5% and 0.7% concentrations of *M. spicata* oil, respectively. **Lane 3**: control, **lanes 4**, **5** and **6**: dead worms after the treatment with 1%, 0.7% and 0.3% concentrations, respectively.

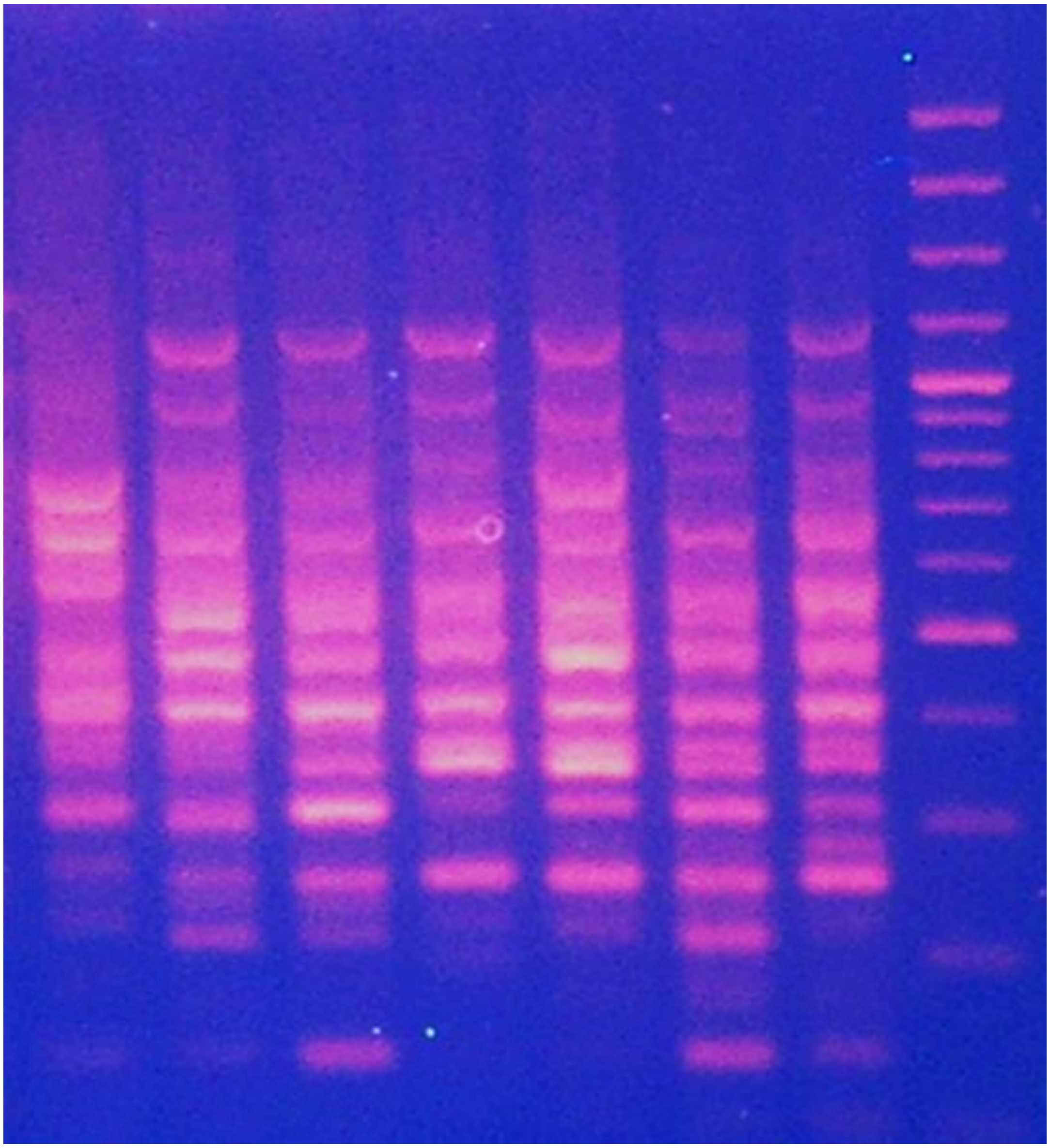

Figure S8. Gel electrophoresis pattern using *PR2* primer. **M**: DNA Marker, **lanes 1** and **2**: alive worms after the treatment with 1.5% and 0.7% concentrations of *M. spicata* oil, respectively. **Lane 3**: control, **lanes 4**, **5** and **6**: dead worms after the treatment with 1%, 0.7% and 0.3% concentrations, respectively.

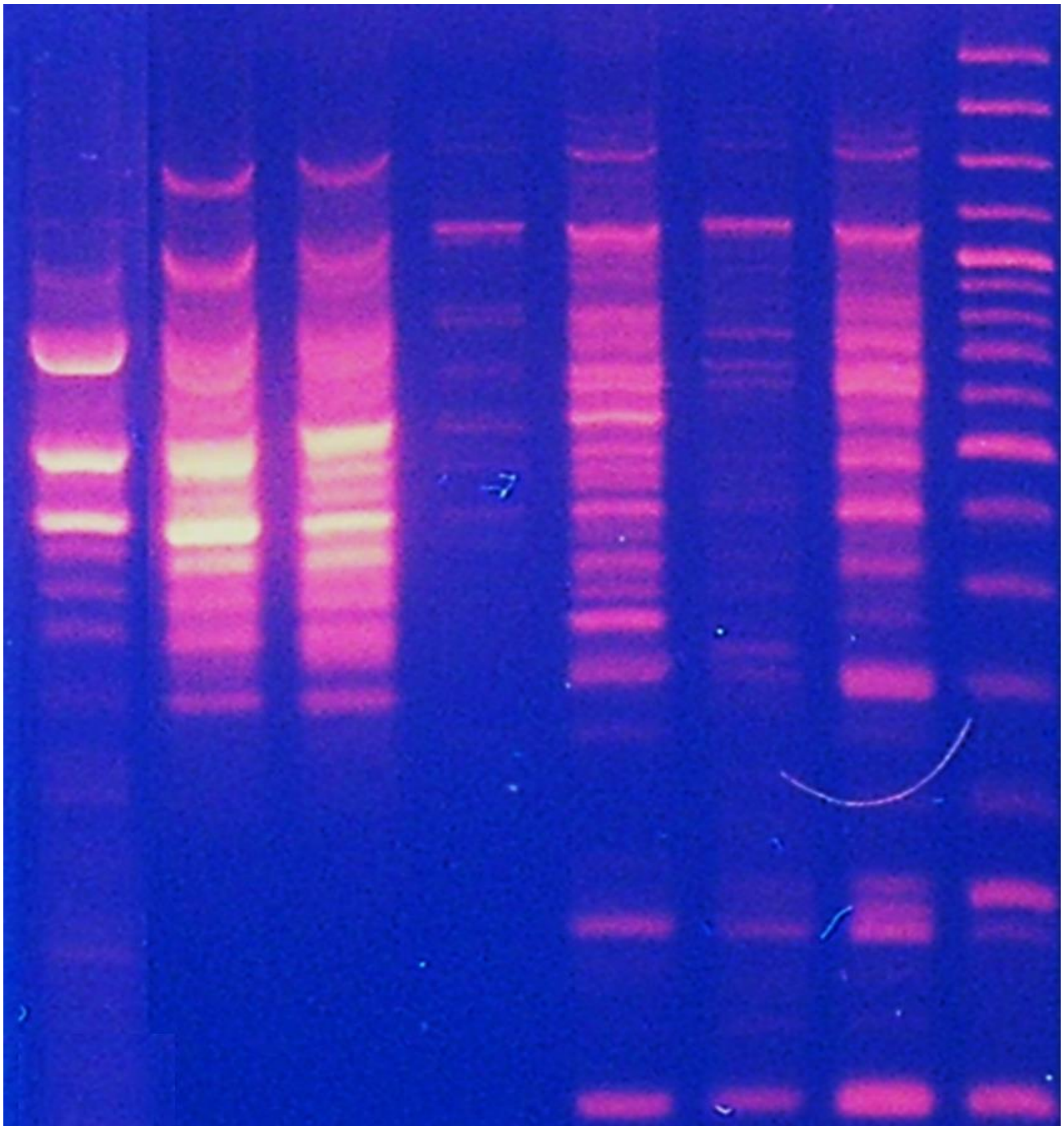

Figure S9. Gel electrophoresis pattern using *Chitenase* primer. **M**: DNA Marker, **lanes 1** and **2**: alive worms after the treatment with 1.5% and 0.7% concentrations of *M. spicata* oil, respectively. **Lane 3**: control, **lanes 4**, **5** and **6**: dead worms after the treatment with 1%, 0.7% and 0.3% concentrations, respectively.
